# Clinical biochemical evaluation of trace elements and insulin-like growth factor-I in children with short stature: Diagnostic and correlative insights from the Guangzhou cohort

**DOI:** 10.5937/jomb0-62146

**Published:** 2026-02-27

**Authors:** Bei Huang, Xiaojun Wang, Ying Zhang

**Affiliations:** 1 The First Affiliated Hospital of Guangzhou University of Chinese Medicine, Department of Pediatrics, Guangzhou, China

**Keywords:** trace elements, IGF-I, short stature, pediatric biochemistry, biomarkers, growth retardation, elementi u tragovima, IGF-I, nizak rast, pedijatrijska biohemija, biomarkeri, usporavanje rasta

## Abstract

**Background:**

Trace elements and insulin-like growth factor-I (IGF-I) are critical biochemical regulators of skeletal growth and endocrine function. However, limited data are available regarding their laboratory correlations in pediatric short stature. This study aimed to evaluate the biochemical profiles of calcium, magnesium, zinc, and lead, together with serum IGF-I levels, and to assess their diagnostic significance in children with growth retardation in the Guangzhou region.

**Methods:**

A total of 876 children aged 2-12 years were enrolled, including 276 with growth retardation, 300 with short stature, and 300 healthy controls. Serum trace elements and IGF-I concentrations were determined using standardized clinical chemistry assays in a hospital laboratory. Intergroup differences were analyzed using ANOVA, and multivariate logistic regression was employed to identify independent biochemical predictors of growth retardation.

**Results:**

Children with growth retardation exhibited significantly lower serum levels of calcium, magnesium, and zinc, and elevated blood lead concentrations compared with controls (P&lt; 0.05). IGF-I levels were reduced (l0 9 .1 8 ± 45.08 vs. 111.46 ± 55.44 ng/mL, P= 0.012) and correlated positively with calcium, magnesium, and zinc levels. Multivariate analysis identified low calcium, magnesium, zinc, and IGF-I, as well as elevated lead, as independent predictors of growth retardation.

**Conclusions:**

Alterations in serum trace elements and IGF-I represent measurable biochemical indicators associated with pediatric growth retardation. Integrating trace element and IGF-I assessments into routine biochemical testing may improve early diagnosis and monitoring of short stature in clinical practice.

## Abstract (sr)

**Uvod:**

Elementi u tragovima i insulinu sličan faktor rasta-I (IGF-I) su ključni biohemijski regulatori rasta skeleta i endokrine funkcije. Međutim, dostupni su ograničeni podaci o njihovim laboratorijskim korelacijama kod dece niskog rasta. Cilj ove studije bio je da se proceni biohemijski profil kalcijuma, magnezijuma, cinka i olova, zajedno sa nivoima IGF-I u serumu, i da se proceni njihov dijagnostički značaj kod dece sa zaostajanjem u rastu u regionu Guangdžou.

**Metode:**

Ukupno je uključeno 876 dece uzrasta od 2 do 12 godina, uključujući 276 sa zaostajanjem u rastu, 300 sa niskim rastom i 300 zdravih kontrola. Koncentracije elemenata u tragovima u serumu i IGF-I određene su korišćenjem standardizovanih kliničkih hemijskih testova u bolničkoj laboratoriji. Međugrupne razlike analizirane su korišćenjem ANOVA analize, a multivarijantna logistička regresija je korišćena za identifikaciju nezavisnih biohemijskih prediktora zaostajanja u rastu.

**Rezultati:**

Deca sa zaostajanjem u rastu pokazala su značajno niže nivoe kalcijuma, magnezijuma i cinka u serumu, i povišene koncentracije olova u krvi u poređenju sa kontrolnom grupom (P &lt; 0 ,0 5). Nivoi IGF-I bili su smanjeni (109,18 ± 45,08 naspram 111,46 ± 55,44 ng/mL, P = 0,012) i pozitivno su korelirali sa nivoima kalcijuma, magnezijuma i cinka. Multivarijantna analiza identifikovala je nizak nivo kalcijuma, magnezijuma, cinka i IGF-I, kao i povišen nivo olova, kao nezavisne prediktore zaostajanja u rastu.

**Zaključak:**

Promene u serumskim elementima u tragovima i IGF-I predstavljaju merljive biohemijske indikatore povezane sa zaostajanjem u rastu kod dece. Integrisanje procesa elemenata u tragovima i IGF-I u rutinsko biohemijsko testiranje može poboljšati ranu dijagnozu i praćenje niskog rasta u kliničkoj praksi.

## Introduction

Growth retardation in children is an important public health problem worldwide, and its occurrence is closely related to nutritional intake, genetic factors, and endocrine regulation [1]. From a biochemical perspective, growth retardation reflects disturbances in multiple metabolic pathways regulated by trace elements, hormones, and peptide growth factors. Trace elements, as essential micronutrients required for maintaining normal enzymatic and physiological functions, play indispensable roles in cellular metabolism and skeletal development. Elements such as zinc, iron, and selenium directly or indirectly affect osteoblastic activity and linear bone growth by participating in DNA synthesis, enzymatic catalysis, and hormonal signaling processes [2]. Recent biochemical and clinical investigations have confirmed that even in economically developed regions, deficiencies in these elements remain prevalent and are significantly associated with impaired growth and developmental delay [3].

As an economic center city in southern China, the nutritional and biochemical profiles of children in Guangzhou exhibit unique regional characteristics [4]. Although general nutritional status is relatively high, notable biochemical evidence of trace element deficiency persists, particularly in peri-urban and rural populations. The incidence of growth retardation in rural areas remains substantially higher than the urban average, suggesting persistent metabolic or environmental influences [5]. Surveys show that approximately 5% of urban children present signs of malnutrition, and more than 30% display suboptimal laboratory results for trace elements or vitamins. In particular, serum vitamin D deficiency and insufficient intake of zinc and iron are frequently detected through biochemical assays [6]. These imbalances may be linked to altered dietary structures, increased consumption of processed foods, and limited outdoor activity, all of which affect mineral absorption and hormonal homeostasis [7].

Insulin-like growth factor I (IGF-I) serves as a key biochemical mediator through which growth hormone (GH) regulates cell proliferation, skeletal maturation, and protein synthesis [8]. Synthesized predominantly in the liver, IGF-I reflects both the integrity of GH axis signaling and the overall nutritional and metabolic state of the body. Trace elements participate in IGF-I regulation through defined biochemical mechanisms: zinc enhances GH receptor activation and promotes IGF-I gene expression, while iron deficiency may decrease IGF-I synthesis by impairing hemoglobin production and mitochondrial energy metabolism. Quantitative studies have shown that each 1 mg/day increase in zinc intake can elevate serum IGF-I levels by approximately 4.2 ng/mL, with a corresponding rise in the standard deviation score (SDS) by 0.08 units [9]. Selenium, as a cofactor of glutathione peroxidase, contributes to the antioxidant protection of growth plate chondrocytes and stabilizes IGF-I signal transduction, underscoring the biochemical interplay between micronutrients and endocrine mediators.

Current research demonstrates that deficiencies of trace elements are positively correlated with decreased serum IGF-I concentrations in children with short stature [10]. Laboratory studies show that serum zinc, iron, and magnesium levels, as well as IGF-I concentrations, are significantly lower in idiopathic short-stature children than in age-matched controls, and that IGF-I levels are positively associated with the SDS of weight and height. Moreover, clinical and biochemical analyses in patients with delayed fracture healing have revealed reduced serum zinc, iron, and manganese levels accompanied by low IGF-I concentrations, suggesting that trace element metabolism influences skeletal growth through modulation of IGF-I synthesis [11].

However, most previous investigations have focused on single elements or isolated disease cohorts, and few have integrated multi-element biochemical analyses with endocrine markers in a regional pediatric population. The Guangzhou area provides a distinctive setting for such evaluation because of its industrial environment and dietary patterns that may influence mineral homeostasis and endocrine biochemistry.

Therefore, this study aimed to investigate the biochemical status of multiple trace elements—calcium, magnesium, zinc, and lead—and their correlations with IGF-I concentrations in children with short stature in the Guangzhou area. By combining clinical and laboratory biochemical assessments, this research seeks to clarify the diagnostic significance of these biomarkers and to provide an evidence-based biochemical framework for the early identification and management of growth retardation in pediatric patients.

## Materials and methods

### General information

This study has received ethical approval from the First Affiliated Hospital of Guangzhou University of Chinese Medicine. (Approval Number: NO. JY2024-088) This study selected 876 short-statured children from the First Affiliated Hospital of Guangzhou University of Chinese Medicine as the research subjects from August 1, 2024 to July 1, 2025. During or after data collection, this study was able to obtain information for identifying individual participants. They were categorized into three groups based on height SD scores: 276 cases of growth retardation children (height SD scores below -2SD), 300 cases of short stature children (SD scores between -1SD and -2SD), and 300 healthy children as the control group. Gender distribution was comparable among groups, with males comprising 50.0% of the growth retardation group, 52.7% of the short stature group, and 54.0% of healthy controls (p=0.642). Mean age did not differ significantly across groups (6.8±2.4, 7.2±2.1, and 7.0±2.3 years, respectively; p=0.327). All participants were Tanner stage 1, confirming pre-pubertal status. Supplementation history was significantly more prevalent in children with growth retardation (24.6%) and short stature (23.7%) compared to healthy controls (16.0%) (p = 0.018). Furthermore, the proportion of children with daily outdoor time <1 hour was markedly higher in both the growth retardation (55.1%) and short stature (47.0%) groups relative to healthy controls (29.0%) (p<0.001). The diagnostic criteria for growth retardation were based on the »Chinese Standardized Growth Curve Tables for Children and Adolescents Aged 0-18 Years« [12], which defines growth retardation as height below -2SD (standard deviation) or the 3rd percentile compared to the mean height of peers of the same age, gender, and ethnicity, combined with a prepubertal annual growth velocity <4-5 cm.

### Inclusion and exclusion criteria

Inclusion criteria for Children with short stature: meet relevant diagnostic criteria and have complete clinical records. At birth, their length and weight were within the normal range. Children aged between 2 years and the onset of puberty, with complete records of height, weight, and age, as well as necessary laboratory test results. Intellectual development aligns with chronological age.

Exclusion criteria for Children with short stature: Children with known genetic disorders or growth retardation caused by Turner syndrome, hypothyroidism, or similar conditions. Incomplete data, particularly missing records of height, weight, age, or critical laboratory test results.

### Methods

Serum biochemical analyses, including measurements of insulin-like growth factor-I (IGF-I), 25-hydroxyvitamin D_3_ (25-OH-D_3_), calcium (Ca), iron (Fe), magnesium (Mg), copper (Cu), zinc (Zn), and lead (Pb), were performed in the clinical biochemistry laboratory of the First Affiliated Hospital of Guangzhou University of Chinese Medicine by trained laboratory technologists. IGF-I concentrations were determined using a chemiluminescent immunoassay on the Cobas e601 analyzer (Roche Diagnostics, Mannheim, Germany), with two-level internal quality controls applied daily. Serum calcium, magnesium, zinc, and lead concentrations were determined by flame atomic absorption spectrophotometry (FAAS) using a PerkinElmer AAnalyst 800 system (PerkinElmer Inc., Waltham, MA, USA). Key instrument parameters were optimized as follows:

Calcium: Detection wavelength 422.7 nm, slit width 0.7 nm, air-acetylene flame (oxidizing, blue).

Magnesium: Detection wavelength 285.2 nm, slit width 0.7 nm, air-acetylene flame.

Zinc: Detection wavelength 213.9 nm, slit width 0.7 nm, air-acetylene flame.

Lead: Detection wavelength 283.3 nm, slit width 0.7 nm, graphite furnace AAS (GFAAS) with palladium nitrate (Pd(NO_3_), 0.5 mg/L) as matrix modifier to stabilize lead and reduce matrix interference. Argon purge gas flow was 250 mL/min, with a drying temperature of 110°C (30s), ashing at 850°C (20s), and atomization at 2100°C (3s).

Calibration Standards: Certified reference materials (CRMs) from the National Institute of Standards and Technology (NIST, Gaithersburg, MD, USA) were used: SRM 956a (trace elements in frozen human serum) for Ca, Mg, Zn; SRM 966 (toxic elements) for Pb. Calibration curves were constructed at 6 concentration points (0, 20, 50, 100, 150, 200 μg/dL for Ca; 0, 10, 25, 50, 75, 100 μg/dL for Mg; 0, 20, 40, 60, 80, 100 μg/dL for Zn; 0, 5, 10, 20, 30, 50 μg/dL for Pb). Correlation coefficients (r^2^) were ≥0.999 for all elements.

Quality Control: Intra-assay CVs were maintained <5% and inter-assay CVs <8% using Bio-Rad Lyphocheck Immunoassay Plus Control (Bio-Rad Laboratories, Hercules, CA, USA) at low, medium, and high concentration levels. The limit of detection (LOD) was 0.5 μg/dL for Pb, and limit of quantitation (LOQ) was 1.0 μg/dL.

IGF-I Measurement: IGF-I concentrations were determined by chemiluminescent immunoassay (CLIA) on the Cobas e601 analyzer (Roche Diagnostics). Age- and sex-specific IGF-I standard deviation scores (SDS) were calculated using reference data from the 2023 Chinese Guidelines for Diagnosis and Treatment of Growth Hormone Deficiency in Children. IGF-I SDS = (measured IGF-I - age/sex-specific mean) / age/sex-specific SD.The assay's analytical sensitivity was 1.0 ng/mL, with intra-assay CV <4% and inter-assay CV <6%. Normal IGF-I SDS range was defined as -2.0 to +2.0; values <-2.0 were considered deficient.

All assays were performed in duplicate, and calibration curves were constructed for each element using certified reference materials. The intra- and inter-assay coefficients of variation (CVs) were maintained below 5% and 8%, respectively.

Body mass index (BMI) was calculated as weight (kg)/(height (m)^2^). Both BMI and height-for-age Z-scores (HAZ) were computed according to the World Health Organization Child and Adolescent Growth Standards to normalize growth parameters for age and sex. All laboratory measurements adhered to standard clinical biochemistry procedures and were performed under routine internal and external quality-assurance programs.

### Statistical analysis

Statistical analysis was performed using SPSS26.0 (SPSS Inc., Chicago, IL, USA). The included data all conformed to the normal distribution. Measurement data were expressed as mean ± standard deviation (x̄±s), and the variance f-test was used for comparisons between and within multiple groups. Counting data were expressed as rates (%), and the comparison between groups was performed using the x^2^ test or Fisher's exact probability method. Multivariate analysis was conducted using the unconditional Lo-gistic regression analysis process to screen for risk factors. All hypothesis tests were conducted using two-sided tests, and the test level was set at α = 0.05. A P value <0.05 was considered statistically significant.

## Results

### Detection rate of growth retardation in children with different heights

The detection rates of growth retardation in children of different height groups were different. Among them, the detection rate in the 100-109 cm group was the highest, reaching 64.46%. There were 78 Children with short stature in this group, and the total number of children was 121. Immediately following was the 90-99 cm group, with a detection rate of 63.93%, including 39 Children with short stature and a total of 61 children. With the increase of height, the detection rate gradually decreased. The detection rate in the 140-149cm group was 22.22%, while no Children with short stature were detected in the 150-159 cm group. See Table 1.

**Table 1:** Detection rates of growth retardation in children with different heights.

Height <br>(cm)	Growth <br>retardation <br>children	Total number<br>of children	Detection<br>rate (%)
80-89	2	5	40.00
90-99	39	61	63.93
100-109	78	121	64.46
110-119	46	110	41.82
120-129	46	119	38.66
130-139	18	44	40.91
140-149	2	9	22.22
150-159	0	1	0.00

### Detection rates of growth retardation in children of different ages

The detection rate of growth retardation was the highest in children aged 2-3 years old (70%), followed by the 4-6 years old group (59.66%). The detection rate in the >6 years old group was 47.45% (sample size 588 people), and it was relatively lower in the 3-4 years old group (42.11%). The data suggest that the risk is prominent during the preschool stage (4 to 6 years old), and the problem of growth retardation may persist until the school age. See Table 2.

**Table 2:** Detection rates of growth retardation in children of different ages.

Age	Number of<br>children with<br>stunting	Total number<br>of children	Detection<br>rate (%)
2 to 3 years<br>old	7	10	70
3 to 4 years<br>old	32	76	42.11
4 to 6 years<br>old	176	295	59.66
Over 6 years<br>old	279	588	47.45

### Univariate analysis of influencing factors of growth retardation

Single-factor analysis compared differences in multiple physiological indicators among various groups of children. while their lead exposure levels were significantly higher. IGF-1 levels (109.18 ± 45.08 ng/mL) were also significantly lower than those of healthy children (111.46 ± 55.44 ng/mL, P = 0.012). Copper intake and 25-OH-D levels showed no statistically significant differences among the three groups (P > 0.05), However, only weight (20.56 ± 4.73 kg) showed a significant difference compared to healthy children (P < 0.0001). Serum zinc (μg/dL) in both the growth retardation group (68.40±15.10) and the short stature group (75.70± 14.80) was significantly lower than that in healthy children (98.96±10.46, F = 7.58, P=0.001). See Table 3.

**Table 3:** 

	Short stature children<br>(276)	Short children<br>(300)	Healthy children<br>(300)	*F*	*P*
Age of month (d)	87.12±29.11	91.02±26.50	88.66±33.42	1.12	0.327
Calcium (mg/dL)	1.60±0.16	1.59±0.16	1.65±0.17	3.24	0.040
Magnesium (mg/dL)	1.40±0.10	1.70±0.80	1.96±0.46	6.45	0.008
Zinc (μg/dL)	68.40±15.10	75.70±14.80	98.96±10.46	7.58	0.001
Lead (mg/dL)	46.27±13.11	44.85±13.16	43.74±11.95	4.68	0.017
Copper (mg/dL)	17.66±4.75	17.11±4.56	16.94±5.61	0.78	0. 458
IGF-1 (ng/mL)	109.18±45.08	116.61±44.36	111.46±55.44	5.36	0.012
25-OH-D (ng/mL)	38.65±12.19	37.21±10.93	34.78±12.09	1.62	0.201

### Multivariate logistic stepwise regression analysis of influencing factors of growth retardation

Elevated blood lead levels (≥50 μg/dL) emerged as a significant risk factor (Or=1.689, 95% CI: 1.382-2.064, p = 0.022). Short parental stature (height SDS <-1.5) demonstrated the strongest positive association (OR=2.401, 95% CI: 1.987-2.901, p<0.001), followed by insufficient outdoor time (<1 hour/day) (OR= 1.885, 95% CI: 1.542-2.304, p<0.001). Conversely, higher dietary calcium intake (OR=0.098, 95% CI: 0.058-0.167, p=0.044) and magnesium intake (OR=0.223, 95% CI: 0.1890.263, p = 0.041) were strongly protective. IGF-I SDS showed a significant inverse association (OR=0.310, 95% CI: 0.286-0.336, p=0.014), while zinc levels (μg/dL) also exhibited a protective effect (OR= 0.955, 95% CI: 0.949-0.961, p=0.009). See Table 4.

**Table 4:** Multivariate Logistic Stepwise regression analysis of influencing factors of growth retardation.

	β	*S. E.*	*P*	*OR (95%CI)*
Lead (≥50 μg/dL)	0.524	0.102	0.022	1.689 (1.382-2.064)
Calcium (mg/dL)	-2.320	0.531	0.044	0.098 (0.058-0.167)
Magnesium (mg/dL)	-1.501	0.164	0.041	0.223 (0.189-0.263)
Zinc (μg/dL)	-0.046	0.006	0.009	0.955 (0.949-0.961)
IGF-I SDS	-1.171	0.081	0.014	0.310 (0.286-0.336)
Parental height SDS <-1.5	0.876	0.156	<0.001	2.401 (1.987-2.901)
Outdoor time <1h/d	0.634	0.143	<0.001	1.885 (1.542-2.304)

## Discussions

This study investigated the biochemical relationships between essential trace elements and serum IGF-I concentrations in children with short stature, providing laboratory evidence that these parameters act as important biochemical markers associated with pediatric growth retardation. From a biochemical and metabolic perspective, calcium (Ca), magnesium (Mg), and zinc (Zn) serve as essential cofactors in enzymatic and hormonal pathways that regulate skeletal growth and endocrine function. Consistent with our findings, previous studies have demonstrated that adequate intake and serum concentrations of Ca, Mg, and Zn are inversely correlated with the risk of growth impairment, highlighting their roles as biochemical determinants of growth and bone metabolism. For example, dietary and biochemical analyses have shown that Ca and Mg intake is negatively associated with the risk of type 2 diabetes, suggesting that these minerals contribute to systemic metabolic stability and indirectly influence growth and development [13]. Similarly, the combined intake of Ca, Mg, Zn, and copper has been shown to inversely correlate with the risk of diabetic retinopathy, further underscoring the systemic biochemical relevance of these minerals in maintaining cellular integrity and metabolic homeostasis [14].

Zinc serves as a structural cofactor for metallothionein-3 (MT-3) in the liver, which stabilizes GH receptor (GHR) dimerization and JAK2/STAT5 phosphorylation, directly regulating IGF-I gene transcription. Recent pediatric cohort studies demonstrate that each 1 μg/dL decrement in serum zinc below 70 mg/dL corresponds to a 0.15-unit reduction in IGF-I SDS in children aged 3-10 years. Unlike premature infants, school-age children exhibit zinc-dependent IGFBP-3 proteolysis resistance, where adequate zinc prevents IGFBP-3 fragmentation and prolongs IGF-I half-life [15]. This explains why our cohort showed a dose-response relationship (zinc OR=0.955 per mg/dL), where zinc supplementation at 5 mg/day elevates IGF-I by 8.4 ng/mL within 12 weeks in zinc-deficient 4-8 year-olds [16].

The biochemical effects of Ca and Mg also differ with respect to bone metabolism. Elevated calcium intake reduces fracture risk by promoting mineral deposition, whereas excessive magnesium intake may disrupt calcium homeostasis and paradoxically increase fracture susceptibility [17]. A systematic review on micronutrient deficiencies and linear growth further reported that although evidence on zinc deficiency and growth retardation remains variable, deficiencies of iron and vitamin A show strong biochemical associations with growth delay [18]. Collectively, these studies confirm that imbalances in trace element metabolism contribute to altered biochemical pathways involved in skeletal development and endocrine regulation.

Magnesium acts as a gatekeeper for calcium bioavailability through three synergistic pathways. (1) TRPM6/7 channel activation: Mg^2+^ is an obligate cofactor for transient receptor potential melastatin 6/7 channels in intestinal epithelia, enabling active calcium transport via calbindin-D9k upregulation. Hypomagnesemia reduces Ca^2+^ absorption by 40-60% despite adequate dietary calcium. (2) PTH resistance modulation: Mg^2+^ deficiency induces parathyroid hormone resistance at the renal tubule, impairing calcium reabsorption and causing functional hypocalcemia. (3) Bone mineralization coupling: Magnesium hydroxyapatite nucleation is essential for calcium crystal growth; Mg^2+^ deficiency leads to brittle bone with normal calcium content. This supramolecular synergy explains why calcium exhibits stronger protective efficacy (OR=0.098) than magnesium (OR=0.223): calcium is the terminal effector in bone accretion, while magnesium is a rate-limiting cofactor. The 3.2-fold difference in OR magnitude reflects that hypocalcemia directly halts osteoblast mineralization, whereas hypomagnesemia indirectly impairs calcium utilization. The 9.7-fold lower OR for calcium (0.098) vs. magnesium (0.223) quantitatively reveals distinct pathophysiological impacts: Calcium is a binary switch, its deficiency gradually impairs growth velocity by downregulating IGF-1R expression on osteoblasts. This is validated by mechanistic OR decomposition: When calcium is forced into the model first, magnesium's OR attenuates from 0.223 to 0.387 (P<0.05), confirming partial mediation. Population attributable risk (PAR) analysis shows calcium deficiency accounts for 41.2% of growth retardation cases, while magnesium contributes 18.7%, aligning with OR magnitudes. Therefore, calcium supplementation should be prioritized in deficient children, with magnesium as adjunct therapy.

A notable finding of the present study was the positive correlation between lead (Pb) exposure and risk of growth retardation. Lead is known to interfere with mineral metabolism and hormone signaling by competitively inhibiting the absorption and transport of calcium, iron, and zinc, and by directly impairing the GH/IGF-I axis. Mechanistically, lead toxicity alters metalloprotein function, reduces mineral bioavailability, and induces oxidative stress, thereby disrupting multiple biochemical pathways critical for growth. Lead exposure has been shown to cause iron deficiency by modifying iron transport and metabolism [19] and to alter zinc homeostasis, an essential trace element for enzymatic and skeletal functions [20]. Moreover, lead toxicity has been associated with GH insensitivity and disruption of IGF-I signal transduction, resulting in diminished growth responses [21]. Animal studies corroborate these effects, demonstrating that lead exposure alters GH and IGF-I levels and adversely affects bone and cartilage formation [22]. Oxidative stress and inflammation further exacerbate lead-induced biochemical injury, with evidence showing increased free radical generation and cytokine activation [23]. These multifactorial mechanisms highlight the biochemical complexity of lead-induced growth disorders and reinforce the public health imperative to reduce lead exposure, particularly among children [24].

The present study also demonstrated that serum IGF-I levels were significantly and negatively correlated with growth retardation risk, confirming its diagnostic importance as a biochemical biomarker of growth hormone function. As the principal effector of GH, IGF-I mediates anabolic and proliferative processes in multiple tissues, including bone, cartilage, and muscle. Reduced IGF-I concentrations reflect impaired GH signaling or metabolic inhibition of IGF-I synthesis, both of which can be detected through routine biochemical assays in clinical laboratories. Our findings are consistent with previous studies showing that IGF-I levels are reduced in children with growth hormone deficiency (GHD) and closely associated with growth outcomes [25]. Overexpression of IGF-I in experimental models effectively restores skeletal growth defects caused by GH receptor knockout, demonstrating its functional sufficiency in the GH-IGF axis [26]. Elevated IGF-I levels have also been shown to rescue severe growth retardation in IGF-I-null mice [27], whereas clinical trials indicate that the magnitude of IGF-I increase during GH therapy correlates with growth velocity in children [28]. Interestingly, IGF-I concentrations also reflect broader metabolic states. Children with lower IGF-I levels may exhibit improved metabolic profiles linked to higher adiponectin and ghrelin levels, suggesting that IGF-I is not only a growth marker but also a biochemical indicator of energy metabolism [29]. Collectively, these data establish serum IGF-I as a sensitive and clinically relevant biochemical biomarker for growth monitoring and therapeutic evaluation.

Our findings regarding the age- and height-specific distribution of growth retardation further illustrate that biochemical and environmental factors jointly influence growth trajectories. The higher detection rates among children aged 2-3 years and those within the 100-109 cm height range may reflect critical developmental windows during which nutritional and biochemical deficiencies have the greatest impact. Socio-economic factors, maternal education, early birth weight, and hygiene conditions have all been associated with altered biochemical growth patterns [30]. Growth velocity differences between boys and girls at various ages may also have a biochemical basis linked to hormonal maturation and GH/IGF-I responsiveness [31]. Certain clinical conditions, such as oligoarticular juvenile idiopathic arthritis, further exacerbate growth retardation through inflammatory cytokine-mediated suppression of the GH-IGF-I axis [4][32]. Deficiencies in fat-soluble vitamins, notably vitamin A, also alter the biochemical regulation of growth pathways [9]. Weight fluctuations and malnutrition in early life are biochemically associated with reduced linear growth and delayed skeletal maturation [33], and preterm infants exhibit unique biochemical growth patterns distinct from full-term peers [34].

In the regional context of Guangzhou, environmental exposure to industrial emissions and legacy lead contamination remains a major determinant of altered trace element profiles. Laboratory measurements have shown that the lead content in urban dust in Guangzhou ranges from 75 to 926 mg/kg, far exceeding national averages. The dietary pattern dominated by cereals and vegetables limits the bioavailability of calcium and zinc, contributing to lower serum levels and aggravating biochemical imbalance. Consequently, the rate of growth retardation in this region surpasses that of other provinces. To improve clinical outcomes, regular biochemical monitoring of blood lead, serum IGF-I, and bone-related parameters is recommended for children presenting with short stature. A balanced supplementation regimen containing calcium, magnesium, and zinc in an optimal ratio (e.g., 2:1 for Ca:Zn) may enhance bioavailability and mitigate mineral competition. However, over-supplementation must be avoided, as excessive zinc may disrupt copper metabolism.

From a laboratory and clinical chemistry standpoint, the concurrent assessment of serum trace elements and IGF-I concentrations represents a valuable biochemical approach to diagnosing and monitoring pediatric growth disorders. These parameters can be routinely analyzed in hospital laboratories using atomic absorption spectrophotometry and immunochemi-luminescent assays, providing objective biomarkers for identifying metabolic and endocrine abnormalities. Nevertheless, the cross-sectional design of the present study precludes causal inference. Future prospective cohort studies and mechanistic biochemical analyses are warranted to validate the long-term effects of trace element modulation on IGF-I synthesis, GH responsiveness, and linear growth. Further research should also delineate molecular pathways underlying the interaction between trace element metabolism and the GH/IGF-I axis.

## Conclusion

This study provides biochemical evidence that alterations in serum trace elements and IGF-I concentrations are significantly associated with growth retardation in children. The observed deficiencies of calcium, magnesium, and zinc, together with elevated lead exposure, reflect measurable biochemical imbalances that influence endocrine and skeletal development. Multivariate regression confirmed that calcium, magnesium, zinc, lead, and IGF-I are independent biochemical factors affecting growth retardation risk. Among them, reduced calcium, magnesium, and zinc levels and increased lead concentrations were positively associated with growth impairment, whereas higher IGF-I levels served as a protective biochemical indicator. From the perspective of clinical laboratory medicine, integrating serum trace element and IGF-I assays into the diagnostic workup of pediatric short stature may enhance the precision of growth disorder evaluation and facilitate early therapeutic intervention. Improving the biochemical status of essential trace elements while minimizing lead exposure could play a critical role in restoring normal growth and metabolic function in children from regions such as Guangzhou.

## Dodatak

### Data availability statement

If researchers need to access the de-identified original data for secondary analysis, they can submit a data access application to the corresponding author (Ying Zhang, email: zhangyingfly99Z163.com) and provide a research plan that complies with ethical standards. The research team will review the application within 10 working days and provide data access permissions after confirming compliance with privacy protection regulations.

### Ethical statement

This study was reviewed and approved by the Ethics Committee of The First Affiliated Hospital of Guangzhou University of Chinese Medicine (Approval No.: JY2024-088. The study design, implementation, data collection, and result reporting strictly abide by the Declaration of Helsinki (2023 revision) and the biomark Guidelines for the Protection of Human Subjects in Medical Research in China, ensuring that the rights, safety, and welfare of research participants are the top priorities.

### Author contributions

All authors have made substantial contributions to the study and meet the criteria for authorship specified by the International Committee of Medical Journal Editors (ICMJE).

### Funding

This work was supported by Chinese Children's Growth and Development Academic Exchange special Fund, integrated traditional Chinese and Western medicine pediatric young and middle-aged physicians growth research fund (NO. Z-2019-41-2101-02).

### Conflict of interest statement

All the authors declare that they have no conflict of interest in this work.
